# Tumor necrosis factor α inhibition overcomes immunosuppressive M2b macrophage-induced bevacizumab resistance in triple-negative breast cancer

**DOI:** 10.1038/s41419-020-03161-x

**Published:** 2020-11-19

**Authors:** Yu Liu, Xuemei Ji, Nannan Kang, Junfei Zhou, Xue Liang, Jiaxin Li, Tianzhen Han, Chen Zhao, Tianwu Yang

**Affiliations:** School of Life Science & Technology, China Pharmaceutical University, Nanjing, 210009 China

**Keywords:** Cancer therapeutic resistance, Breast cancer

## Abstract

Bevacizumab in neoadjuvant therapy provides a new hope of improved survival for patients with triple-negative breast cancer (TNBC) by targeting vascular endothelial growth factor in combination with chemotherapy, but curative effect is limited by bevacizumab’s continuous use while mechanisms remain incompletely understood. More and more researches reported that tumor-associated macrophages mediate resistance to chemotherapy and radiotherapy in various tumors. Here we developed a TNBC model resistant to bevacizumab under bevacizumab continuous administration. It was found that proportion of a specific subset of tumor-associated macrophages characterized as M2b (CD11b^+^ CD86^high^ IL10^high^) increased and responsible for acquired resistance to bevacizumab. Then, we showed that RAW264.7 macrophages could be polarized to M2b subtype on simultaneous exposure to bevacizumab and TLR4 ligands as occurs in the context of continuous bevacizumab treatment. Concordantly, in TLR4-deleted C57BL/10ScNJNju (TLR4^lps–del^) ^*mut/mut*^ mice with bevacizumab treatment model, it was verified that the M2b macrophage could be induced by Fc gamma receptor-TLR4 cross-talk. In MDA-MB-231-resistant tumor-bearing mice, the content of TNFα in serum kept going up consistent with CCL1, a chemokine of M2b macrophage. In vitro neutralizing tumor necrosis factor α (TNFα) could inhibit the tumor progression caused by M2b culture medium and tumor IDO1 expression. Therefore, we thought that TNFα is a key tumor-promoting effector molecule secreted by M2b macrophage. Accordingly, the curative effect of bevacizumab was proved to be significantly improved by neutralizing TNFα with anti-TNFα nanobody. This study is expected to provide theoretical and clinical evidence elucidating the drug resistance in patients receiving bevacizumab.

## Introduction

Bevacizumab is a recombinant humanized monoclonal IgG1 antibody that binds to vascular endothelial growth factor and inhibits the proliferation of endothelial cells and the formation of new blood vessels^1^. In the latest updated medical guideline of bevacizumab by European Medicine Agency, bevacizumab is approved for recurrent or metastatic breast cancer by in combination with paclitaxel and capecitabine. It is especially applied for patients who has a high tumor burden or rapidly progressive disease and human epidermal growth factor receptor 2-negative, and hormone receptor-negative or hormone receptor-positive but refractory to endocrine therapy. Bevacizumab has been shown to increase rate of pathological complete response in several clinical trials^2–4^. Despite a statistically significant increase in progression free survival, studies demonstrated no major benefits to overall survival was described and patients inevitably relapsed owing to acquired resistance^5^. The induction of macrophage migration inhibitory factor^6^, and tyrosine kinase c-Met^7^ in tumor cells and for host cell-mediated resistance, the involvement of tumor-associated macrophages (TAM), myeloid-derived suppressor cells, vascular pericytes and Fibrocyte-like cells^8^ mediate acquired resistance to bevacizumab has been reported in mice. Nowadays, the mechanism for bevacizumab resistance has been studied in non-small cell lung cancer^9^, glioblastoma^10^, and ovarian cancer^11^. However, the efficacy and mechanisms of bevacizumab on intractable triple-negative breast cancer (TNBC) has not be reported. The effect evaluation and mechanism research of longtime and continuous bevacizumab treatment for TNBC is urgently to be needed.

Increased TAMs within the tumor microenvironment play significant roles in cancer progression and dissemination^12,13^. TAMs are myeloid-derived mononuclear cells with certain phenotypic features, such as CD11b^+^, CD68^+^, and F4/80^+^, and display remarkable plasticity under different micro-environmental stimuli. Proinflammatory M1 macrophages are characterized as IL10^low^ IL12^high^ CD86^+^ MHCII^+^, are classically activated by LPS or IFNγ^12^, whereas anti-inflammatory M2 macrophages can be subdivided into four heterogeneous subtypes distinguished from each other by a series of hallmarks and cytokines: M2a, M2b, M2c, and M2d^14^. M2b macrophages (IL10^high^ IL12^low^ CD86^+^ MHCII^+^), whose characteristics is between M1 classically activated macrophages with proinflammatory antitumoral function and M2 alternatively-activated macrophages and promote tumor growth and invasion, are induced by combined immune complexes as well as TLR or IL-1R agonists and produce IL1, IL6, IL10 and TNFα^1,15,16^. It has been reported M2b macrophage promote tumor by releasing immunosuppressive cytokine IL10^17^, and proinflammatory cytokine leads to inflammation correlated with tumor development^18^. The mechanisms of M2b in various functions remain largely speculative.

TNFα (Tumor necrosis factor α) is considered to induce tumor necrosis with a megadose^19^, whereas it plays a significant accelerative impact upon tumors and plays multiple roles in the immune response of the tumor microenvironment^20,21^, cell proliferation and invasion^22^, and angiogenesis^23,24^. Inhibition of TNFα significantly improved the efficacy of MAPK inhibitors in treatment of melanoma^25^. The relationship between TNFα and immunosuppression is linked through indoleamine 2,3 dioxygenase 1 (IDO1), with TNFα involved in facilitating accumulation of IDO1^26^. The immunosuppressive molecule IDO1 is the rate-limiting enzyme in the metabolic pathway that converts tryptophan to kynurenine^27,28^. IDO1 is upregulated in many cancers and is a predictor of immune escape^29,30^.

Here, we developed a tumor transplantation model under bevacizumab continuous administration. We proved M2b macrophage cells, defined as CD86^+^, CD11b^+^, and IL10^high^, contribute to the acquired resistance to bevacizumab, and clarified that M2b cells were induced and polarized through FcγRs cross-talking with TLR4 signaling pathway in vitro and in vivo. We further discovered the resistance effect produced by M2b type macrophage through TNFα secretion and IDO1 activation. Finally, we hypothesized that the inhibition of TNFα might reverse bevacizumab resistance and proved the effectiveness. All research results are essential to understand how bevacizumab resistance develops and how it may be overcome in TNBC.

## Materials and methods

### Animal experiments

Mice were purchased from Model Animal Research Center of Nanjing University (Nanjing, China) and kept under the pre-approved guidelines of the Animal Care and Use Committee of China Pharmaceutical University (Nanjing, Jiangsu, China). MDA-MB-231 and 4T1 breast cancer cells were purchased from the American Type Culture Collection (Manassas, VA, USA). E0771 breast cancer cells were purchased from Fu Heng Biology (Shanghai, China). Female BALB/c mice (6–8 weeks old) were injected subcutaneously in the left flank with 2×10^5^ 4T1 cells or 1×10^7^ MDA-MB-231 cells (*n* = 5–7). Treatment with anti-TNFα nanobody (10 mg/kg i.p. once daily) or bevacizumab (10 mg/kg i.p. once weekly) or both began on day 7–15 after tumor injection via intraperitoneal injection. Female C57BL/10ScNJNju *(TLR4*^lps–del^*)*^*mut/mut*^ and wild-type C57BL/10ScNJGpt mice (6–8 weeks old) were injected subcutaneously with 2 × 10^6^ E0771 cells in the right flank. Treatment with bevacizumab, the F(ab′)_2_ of bevacizumab, or PBS was initiated when tumor become apparent. Bevacizumab (10 mg/kg i.p. once weekly) or the F(ab′)_2_ region of bevacizumab (7.3 mg/kg i.p. daily) and PBS were administered via intraperitoneal injection for 1 month.

### Real-time PCR analysis and primers used for qPCR analysis

Total RNA was extracted and reverse transcribed with HiScript II Q Select RT Super Mix for qPCR (Vazyme Biotech, Nanjing, China). Quantitative real-time (RT-PCR) was performed by CFX96™ and CFX384™ Real-Time PCR Detection Systems (Bio- rad, Hercules, CA, USA). Relative quantification of mRNA levels was conducted using the comparative Ct method with β-actin as the reference gene and the formula 2^−ΔΔCt^. Primers used for qRT-PCR were downloaded from Primer Bank (https://pga.mgh.harvard.edu/primerbank/).

### Flow cytometry staining and analysis

Cells from tumors or dishes were stained with fluorescein isothiocyanate-conjugated anti-CD86 (561962; BD Biosciences, Franklin Lakes, NJ, USA), PE-conjugated anti-CD11b (101207; BioLegend, San Diego, CA, USA), APC-conjugated anti-IL10 (561059; BD Biosciences), Alexa Fluor 647-conjugated anti-IDO1 (654003; BioLegend) and fluorescence-activated cell measuring was performed with a Accuri C6 plus (BD Biosciences). Data were analyzed with the flow cytometry analysis program FlowJo V10.4.

### Immunohistochemistry staining

The sections were incubated overnight with primary antibodies against CD206 (ab64693; Abcam, Cambridge, MA, USA), CD86 (ab119857; Abcam), CD68 (ab31630; Abcam), TNFα (ab1793; Abcam), IL10 (ab189392; Abcam), or HMGB1 (ab79823; Abcam); afterwards, they were incubated with secondary antibodies: goat anti- rabbit IgG H&L (ab150077; Abcam) goat anti-mouse IgG H&L (ab150117; Abcam), or goat anti-rat IgG H&L (ab150167; Abcam). The slides were then washed and treated with diaminobenzidine (DAB) chromogen for 3–5 min, yielding a dark brown color. The sections were counterstained with hematoxylin and scanned at ×40 using a Nano Zoomer 2.0 HT (Hamamatsu Photonics K.K., Hamamatsu, Japan).

### Macrophage polarization in vitro

RAW264.7 cells were stimulated with lipopolysaccharide (LPS; 20 ng/ml; Sigma-Aldrich, St. Louis, MO, USA) alone or a mixture with bevacizumab (250 μg/ml; Roche, Basel, Switzerland), human IgG (250 μg/ml, Solarbio, Beijing, China), F(ab′)_2_ of bevacizumab (250 μg/ml), or antibodies alone (250 μg/ml) for 48 h. Cells and medium from each group were collected and cell were harvested with a cell scraper after the stimulus was completed.

### Transwell assay

RAW264.7 cells were seeded into 24-well plates and the medium was replaced with fresh medium containing LPS (20 ng/ml), bevacizumab (250 μg/ml), human IgG or F(ab′)_2_ of bevacizumab, a mixture of antibody and LPS after 24 h. Then, 4T1 cells were seeded in the upper chambers of transwell plates. As for TNFα neutralization, anti-TNFα-nanobody was added to the lower chambers of transwell plates, which were maintained at 37 °C under 5% CO_2_ for 24 h. The cells were then washed and fixed with 4% paraformaldehyde (Solarbio, Beijing, China) for 30 min. The fixed cells were stained with 0.1% crystal violet (Sinopharm Chemical Reagent Co., Ltd, Beijing, China) for 30 min and photographed using a microscope. The same microscope was used to photograph different views of the stained monolayer.

### Statistical analyses

The data are presented as means ± s.d. using GraphPad Prism 7.0. All experiments were repeated at least three independent times. Statistical analysis was performed using GraphPad Prism 7.0. *P* values < 0.05 were considered statistically significant.

## Results

### Acquired resistance to bevacizumab in mouse models accompany with changed cytokines pattern

**Fig. 1:**
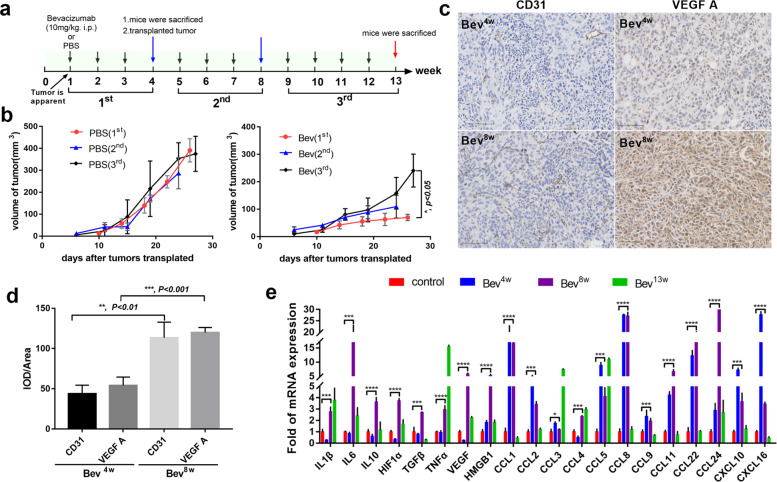
Continuous treatment with bevacizumab induces acquired resistance and cytokines change in vivo. **a** Schematic of experimental design for bevacizumab resistance model development. **b** Tumor growth volume over time following subcutaneous transplant of tumor tissues into Balb/c nude mice. *n* = 5 for each condition. *P < 0.05 by two-way ANOVA test. Error bars represent s.d. **c** Representative images of sections from tumors at the time points of 4-week and 8-week stained for CD31 and VEGF A. Scale bar, 100 μm. **d** Quantitative evaluation of CD31 and VEGF A in each tumor of mice (IOD/Area). **P < 0.01; ***P < 0.001 by *t* test. Error bars represent s.d. **e** qRT-PCR of IL1β, IL6, IL10, HIF1α, TGFβ, TNFα, VEGF, HMGB1, CCL1, CCL2, CCL3, CCL4, CCL5, CCL8, CCL9, CCL11, CCL22, CCL24, CXCL10, CXCL16 mRNA levels in tumor tissues of 4-week, 8-week and 13-week. ***P < 0.001; ****P < 0.0001 by *t* test. Error bars represent s.d.

To develop the model of acquired resistance to bevacizumab, breast cancer xenograft bearing mice were treated with bevacizumab until tumor became aggressive, when tumor tissue was transplanted, and finally tumors were collected at 13 weeks (Fig. 1a). As expected, bevacizumab treatment effectively suppressed the tumor growth at first 4 weeks compared with the control. Interestingly, in 5–8 weeks and 9–13 weeks, tumor accelerated progression despite continuous bevacizumab treatment (Fig. 1b). We evaluated the CD31 and VEGF A bevacizumab or PBS-treated mice at time point of 4-week, 8-week. Compared with time point of 4- week, there is a significant increase in CD31 and VEGF A in 8-week points under bevacizumab treatment (Fig. 1c–d), confirming a resistant phenotype in these tumors. Next, we asked whether the cytokines, and chemokines, expressed in or secreted from tumors changed, qRT-PCR showed changed cytokines profiles in tumors of bevacizumab resistance (Fig. 1e). Thus, those results indicate that bevacizumab resistance in mouse models accompany with changed cytokines pattern.

### CD11b^+^ CD86^+^ IL10^+^ tumor-associated macrophage was dominate subtype in tumor tissue after bevacizumab continuous treatment

**Fig. 2:**
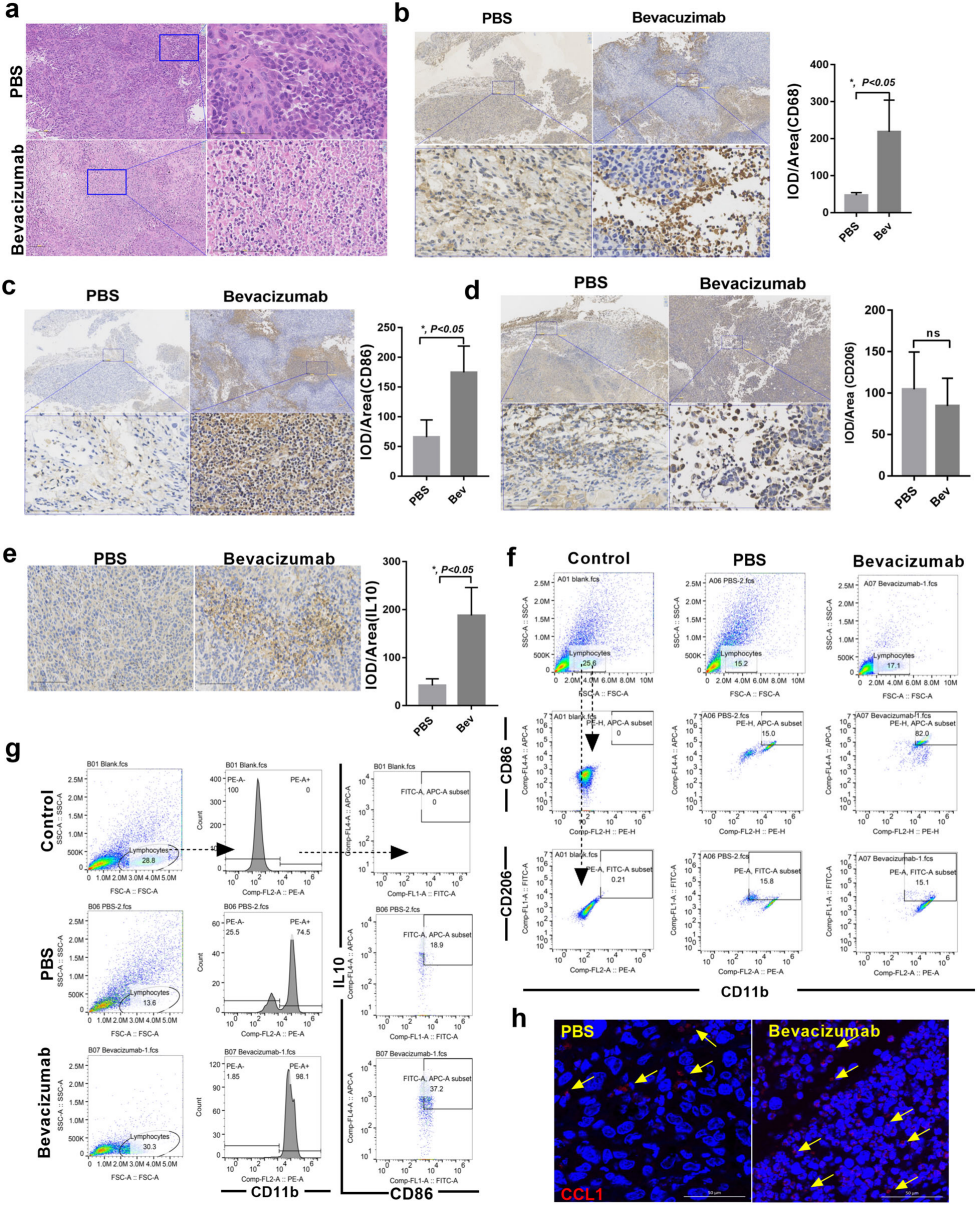
CD11b^+^ CD86^+^ IL10^+^ tumor-associated macrophage was dominate subtype in tumor tissue after bevacizumab continuous treatment. **a** Representative H&E staining image of tumor sections from mice treated with bevacizumab and PBS at 8-week. Scale bar, 100 μm. **b** Representative image and quantitative evaluation of tumor sections from mice treated with bevacizumab and PBS stained for CD68 in each tumor of mice at 8-week (IOD/Area). *n* = 5. Scale bar, 100 μm. *P < 0.05 by *t* test. Error bars represent s.d. **c** Representative image and quantitative evaluation of tumor sections from mice treated with bevacizumab and PBS stained for CD86 at 8-week. Scale bar, 100 μm. *P < 0.05 by *t* test. Error bars represent s.d. **d** Representative image and quantitative evaluation of tumor sections from mice treated with bevacizumab and PBS stained for CD206 at 8-week. Scale bar, 100 μm. *P < 0.05 by *t* test. Error bars represent s.d. **e** Representative image and quantitative evaluation of tumor sections from mice treated with bevacizumab and PBS stained for IL10 at 8-week. Scale bar, 100 μm. *P < 0.05 by *t* test. Error bars represent s.d. **f** Macrophages were detected as CD11b^+^ CD86^+^ or CD11b^+^ CD86^+^ cells, respectively. Each sample contained tumor tissues from three mice. **g** Representative analysis of the single-cell suspended from PBS or bevacizumab-treated tumor tissues. Macrophages were detected as CD11b^+^ CD86^+^ IL10^+^ cells. Each sample contained tumor tissues from three mice. **h** Representative images of double staining for CCL1 and nucleus. Scale bar, 50 μm.

To determine the relationship between changed cytokines and tumor-associated macrophage in bevacizumab resistance model because macrophages are major source of cytokines and always involved in anti-angiogenic resistance in tumor therapy^12,31^. We examined the macrophages infiltration in tumors at 8-week when tumor acquired resistance to bevacizumab, whereas CD68^+^ CD206^+^ for classic M2 macrophage and CD68^+^ CD86^+^ for M1 macrophage. H&E staining suggested that monocyte, which characterized as irregular nucleus aggregated in bevacizumab-treated tumors (Fig. 2a). CD68^+^ macrophages were upregulated in bevacizumab-treated tumors (Fig. 2b). We next examined phenotypical CD86^+^ or CD206^+^ macrophages and found CD86^+^ cells were significantly increased in the bevacizumab-resistant section (Fig. 2c) but not CD206^+^ cells (Fig. 2d). As IL10 was highly secreted by M2 macrophages but hardly expressed in M1 macrophages. We further detected IL10 expression in PBS or bevacizumab-treated tumors and surprisingly found IL10 was highly expressed in bevacizumab-treated tumor (Fig. 2e). These data show CD68^+^ or/and CD86^+^ monocyte was increased in bevacizumab resistance tumor.

To gain insight into the macrophages type in bevacizumab resistance tumor further, the characterized CD11b^+^ CD86^+^ or CD11b^+^ CD206^+^ macrophages in suspensions of tumor tissues were detected by flow cytometry. There was no significant difference between the CD11b^+^ CD206^+^ macrophages in tumors treated with PBS and bevacizumab, but macrophages in the PBS-treated group only expressed CD11b^+^ CD86^+^ in a small number of macrophages (15%), whereas 82% of bevacizumab-resistant group (Fig. 2f). IL10 was also used to count the cells and CD11b^+^ CD86^+^ IL10^high^ macrophages increased in mice treated with continuous bevacizumab (Fig. 2g). Thus, by comparing with the phenotypic characteristics of macrophages, we found CD11b^+^ CD86^+^ IL10^high^ macrophages is defined as M2b macrophages, a non-canonical M2 macrophage^15^. So we also analyzed CCL1, which is secreted by M2b cells specifically^18^, and we observed that the additional macrophages present were CCL1^+^ cells in the bevacizumab treatment group (Fig. 2h). We performed enzyme-linked immunosorbent assay (ELISA) (Supplementary Fig. 1a) and tube formation assay (Supplementary Fig. 1b) to ensure the bevacizumab could neutralize mouse VEGF. Overall, these results indicate that the major change engendered by bevacizumab therapy, was to increase the ratio of M2b macrophages compared with control, which may be a part of reason why cytokines pattern changed in bevacizumab resistance tumor.

### Macrophage polarize to M2b subtype dependent on TLR4 and bevacizumab induced Fc gamma receptor co-activation

**Fig. 3:**
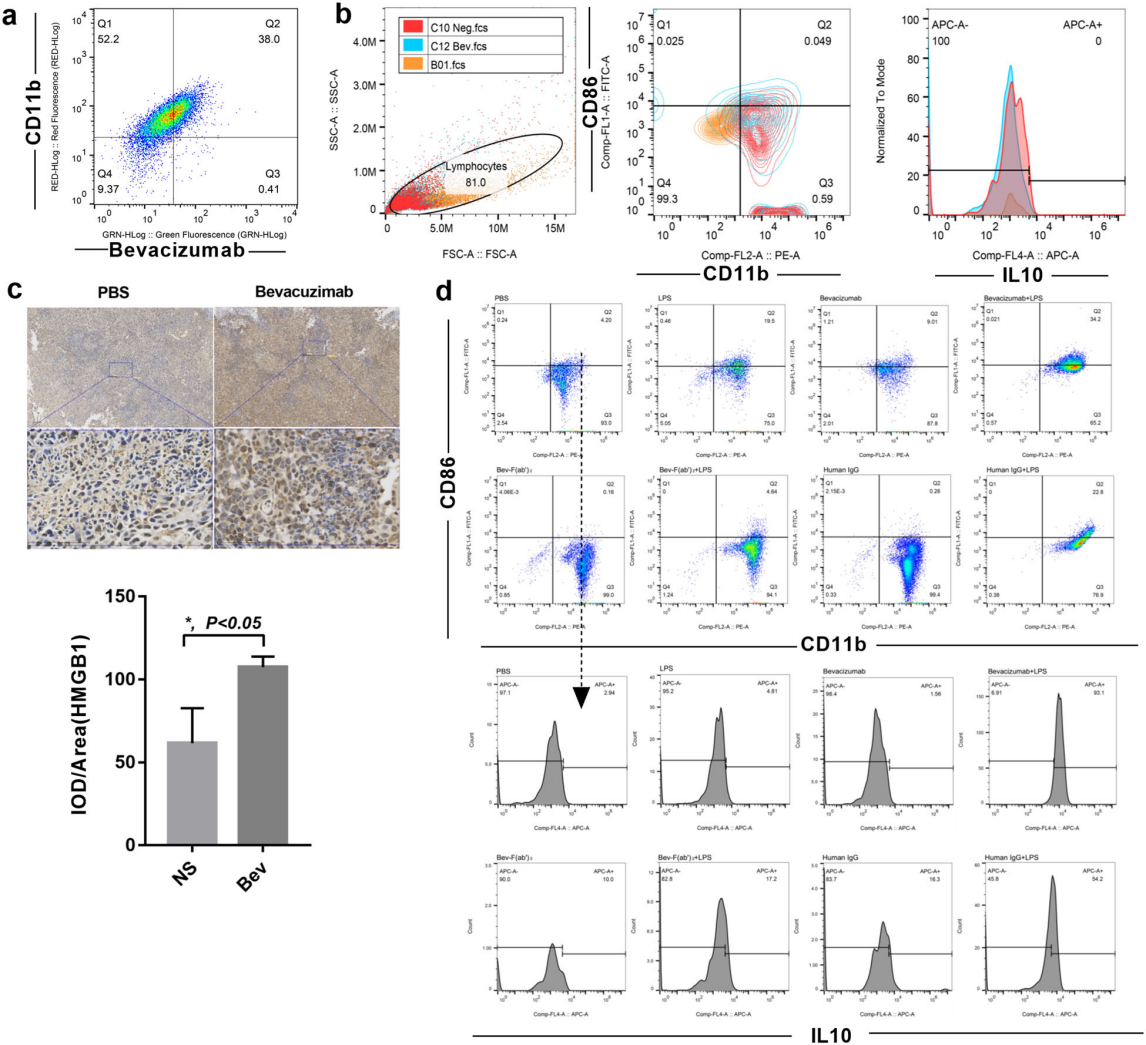
In the presence of bevacizumab and TLR4 agonists, macrophages polarize to the M2b subtype in vitro. **a** Representative analysis of the bevacizumab binding mouse macrophages (bevacizumab-FITC, CD11b-PE). **b** Flow cytometry analysis of CD86, CD11b and IL10 expression in macrophages treated with bevacizumab and PBS respectively. (CD11b-PE, CD86-FITC, IL10-APC). **c** Representative image and quantitative evaluation of tumor sections from mice treated with bevacizumab and PBS stained for HMGB1 at 8-week. Scale bar, 100 μm. *P < 0.05 by *t* test. Error bars represent s.d. **d** Analysis of the RAW264.7 macrophages treated with bevacizumab, F(ab′)_2_ of bevacizumab, human IgG, alone or in combination with LPS for 48 h. Cells were stained for CD11b, CD86, and intracellular IL10.

To address why M2b macrophages increased under bevacizumab stimulation in vivo, we treated RAW264.7 macrophages with or without bevacizumab. First, we demonstrated that the bevacizumab can bind to mouse macrophages (Fig. 3a). To mimic the bevacizumab interaction with macrophages in animal, we exposed RAW264.7 macrophages in bevacizumab and detected the phenotypic changes. There was no difference between bevacizumab and PBS-treated RAW264.7 cells (CD11b^+^ CD86^low^ IL10^low^) (Fig. 3b), which indicates that bevacizumab alone could not induce macrophage polarization to the M2b subtype. The analyses evaluated the cytokines secretion of TNFα, IL6, and IL10 in Fig. 1e, those three cytokines also highly expressed in M2b macrophages, and could be upregulated via activation of TLR4/IL-1R induced NK-κB pathway^32^. As an endogenous ligand of toll-like receptor 4, HMGB1 was largely generated in the bevacizumab administered tumors at 8-week (Fig. 1e, 3c). We hypothesized that M2b macrophage induction in xenografts after bevacizumab treatment was mediated by co-activation of FcγR and TLR4. A simplified in vitro model of M2b macrophage polarization was established. LPS was TLR4 activator while M0 macrophage polarize to M1 subtype (CD11b^+^ CD86^+^ IL10^low^) under LPS stimulation^33^. We stimulated RAW264.7 cells with bevacizumab, F(ab′)_2_ of bevacizumab, human IgG each alone or combined with LPS and then detected macrophage phenotype, and found only combine LPS and bevacizumab or human IgG induced RAW264.7 cells polarize to CD11b^+^ CD86^+^ IL10^high^ macrophage, consistent with enhanced under bevacizumab administration in vivo (Fig. 3d). These demonstrated that in the presence of bevacizumab and TLR4 agonists, macrophages polarize to the M2b subtype in vitro.

**Fig. 4:**
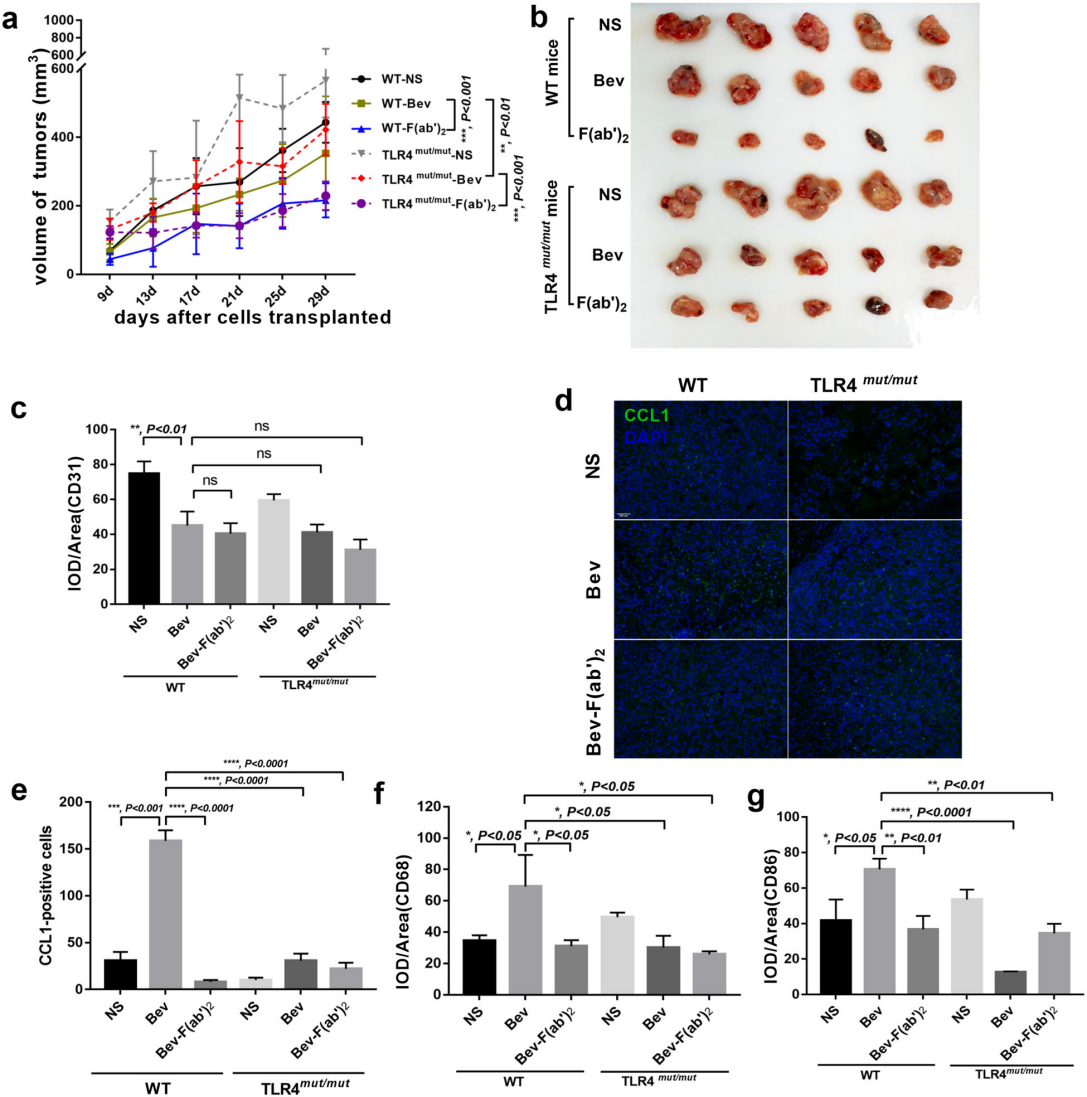
TLR4 and FcγR signaling were responsible for regulating M2b macrophages in vivo. **a**–**b** Tumor growth volume and photos over time following subcutaneous transplant of tumor tissues into wild type and (TLR4^lps–del^) ^*mut/mut*^ C57BL/10 mice. *n* = 5 for each condition. **P < 0.01; ***P < 0.001 by two-way ANOVA test. Error bars represent s.d. **c** Quantitative evaluation of CD31 in each tumor of mice (IOD/Area). **P < 0.01 by *t* test. Error bars represent s.d. **d**–**e** Representative images and quantitative evaluation of double staining for CCL1 and nucleus. Scale bar, 200 μm. ***P < 0.001; ****P < 0.0001 by *t* test. Error bars represent s.d. **f** Quantitative evaluation of CD68 in each tumor of mice (IOD/Area). **P < 0.01 by *t* test. Error bars represent s.d. **g** Quantitative evaluation of CD86 in each tumor of mice (IOD/Area). **P < 0.01 by *t* test. Error bars represent s.d.

Then we subcutaneously injected C57BL/10ScNJNju *(TLR4*^lps–del^)^*mut/mut*^ and wild-type C57BL/10ScNJGpt mice with mouse TNBC cells E0771 and treated tumors with bevacizumab, the F(ab′)_2_ of bevacizumab, or normal saline, referring to the pharmacokinetics of lgG, F(ab′)_2_, and Fab′ treatment in mice^34^. As expected, treatment with bevacizumab and its F(ab′)_2_ inhibited tumor growth (Fig. 4a–b) and angiogenesis (Fig. 4c) compared with the control. Interestingly, F(ab′)_2_ was more effective at treating wild-type mice than was bevacizumab (Fig. 4a). We subsequently examined CCL1 (Fig. 4d–e), total macrophages (CD68^+^, Fig. 4f), and CD86 (Fig. 4g), all of which suggested that M2b macrophages had decreased in *TLR4* ^*mut/mut*^ tumors compared to levels in wild-type mice as well as in those treated by F(ab′)_2_ compared with those treated with bevacizumab. Collectively, the data indicated that TLR4 and FcγR signaling were responsible for regulating M2b macrophages.

### M2b macrophages-TNFα-IDO1 axis induce tumor progression in vitro

**Fig. 5:**
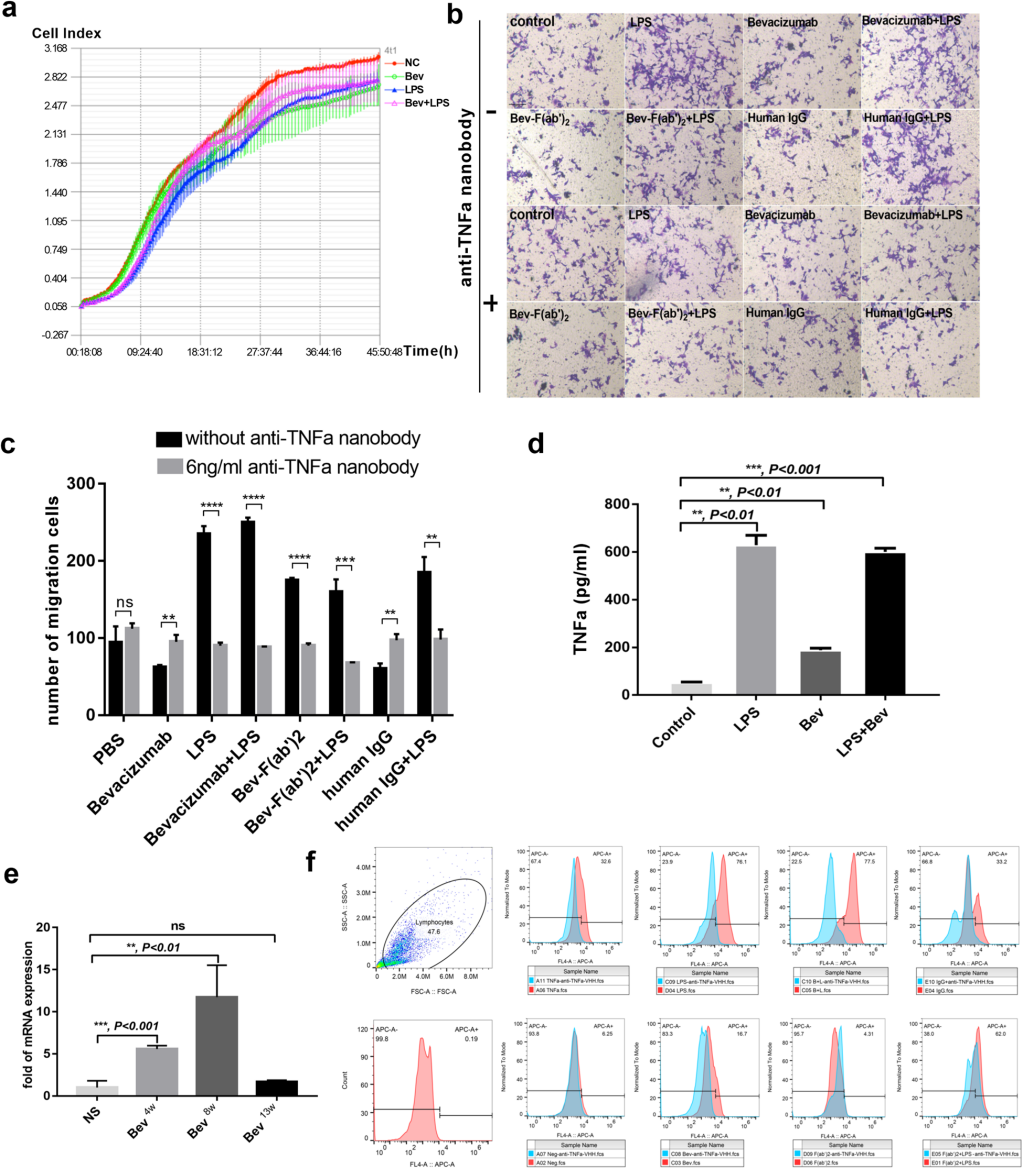
M2b macrophages inducing tumor cell migration and IDO1 expression through TNFα in vitro. **a** Real-time cell analysis curve of 4T1 cell incubated with different RAW264.7 macrophage conditional medium (NC as control, Bev, bevacizumab). **b** Representative transwell migration images of 4T1 attracted by conditioned medium from different RAW264.7 macrophage. Scale bar, 200 μm. **c** Quantitative evaluation of number of migrated cells in **b**. *n* = 3. **P < 0.01; ***P < 0.001; ****P < 0.0001 by *t* test. Error bars represent s.d. each transwell assay were independently repeated three times. **d** ELISA for analyzing the level of secreted TNFα in RAW264.7 cells under bevacizumab, either alone or in combination with LPS treatment. *n* = 3. **P < 0.01; ***P < 0.001; ****P < 0.0001 by *t* test. Error bars represent s.d. **e** qRT-PCR of IDO1 mRNA levels in tumor tissues of 4-week, 8-week, and 13-week. *n* = 3. **, *P* < 0.01 by *t* test. Error bars represent s.d. **f** Flow cytometry analysis of intracellular IDO1 expression in 4T1 cells incubated with different RAW264.7 macrophage conditional medium added 6 ng/ml anti-TNFα nanobody or not.

To investigate the potential role of M2b macrophages in tumor cell progression, we cultured tumor cells with cellular supernatant of M0, M2b, M1 macrophage, and detected tumor proliferation with real-time cell analysis, but there was no change among 4 T1 cells treated by four kinds of cellular supernatant (Fig. 5a). Next, we performed transwell assay to evaluate 4T1 cells affected by M2b macrophage. Indeed, the LPS or LPS combined with antibodies facilitated migration of 4T1 cells (Fig. 5b–c). As M2b macrophages present a TNFα^high^ feature and our previous study demonstrated that the anti-tumor effects of docetaxel are strengthened through neutralization of the proinflammatory cytokine TNFα^35^. Furthermore, we found that TNFα maintained sustainable growth sharply after tumor became resistant to bevacizumab (Fig. 1e). To examine whether TNFα induce 4T1 cell migration, TNFα was neutralized by anti-TNFα nanobody and then migration of 4T1 cells was inhibited (Fig. 5b–c). Thus, we measured the production of TNFα in culture medium by ELISA after RAW264.7 cells were polarized to the different subtype. Compared with control, M2b macrophages produced large quantities of TNFα (601.28 ± 14.87 pg/ml) (Fig. 5d). These data demonstrate that M2b macrophages promote tumor metastasis via TNFα.

A previous study by Banzola et al.^26^ indicated that TNFα treatment induced IDO1 expression and release in prostate cancer cell lines. To assess whether immunosuppressive molecule IDO1 expression was induced upon exposure to M2b macrophages, we first analyzed IDO1 during tumors acquired bevacizumab resistance in vivo by qRT-PCR (Fig. 5e) and found IDO1 expression in a high level at 8-week. We stimulated 4T1 breast cancer cells with conditional medium of M2b macrophages and control medium, and the results showed that IDO1 was remarkably increased in TNFα enriched conditional medium. We then added anti-TNFα nanobody to the conditional medium and found that IDO1 expression was decreased in M2b and M1 macrophage conditional medium group (Fig. 5f). These results suggest that M2b macrophages upregulated IDO1 via TNFα.

### The TNFα in serum depends on occurrence of bevacizumab resistance

**Fig. 6:**
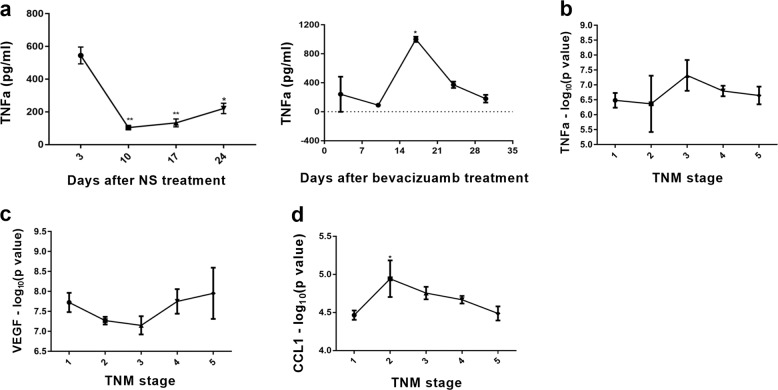
TNFα increased in bevacizumab resistance tumors. **a** Serum concentration-time curve of TNFα in MDA-MB-231R bearing mice treated with bevacizumab or saline was detected by ELISA. *n* = 3/group. *P < 0.05; **P < 0.01 by *t* test. Error bars represent s.d. **b** TNFα expression levels in breast cancer patients treated with bevacizumab at different clinical stages. Error bars represent s.d. **c** VEGF expression levels in breast cancer patients treated with bevacizumab at different clinical stages. Error bars represent s.d. **d** CCL1 expression levels in breast cancer patients treated with bevacizumab at different clinical stages. Error bars represent s.d. *P < 0.05 by *t* test.

TNFα is capable of inducing angiogenesis and tumor metastasis^36^. To get insight into TNFα has relevance to bevacizumab resistance in more detail in in vivo, we separated MDA-MB-231 cell lines resistant to bevacizumab (MDA-MB-231R) and injected MDA-MB-231R on one flank of mice with or without bevacizumab administration, then detected TNFα concentration in serum of tumor-bearing mice every week (*n* = 4). The tumor progressed rapidly (data not shown) and we stopped bevacizumab when mice were in poor shape. We found that the concentration of TNFα increased with bevacizumab treatment and decreased when we stop drug administration because bevacizumab therapy was ineffective (Fig. 6a), which showed a trend similar to TNFα changes in different TNM stage of clinical samples’ microarray (GSE103668) (Fig. 6b). We further analyzed VEGF and CCL1 in clinical samples’ microarray, whose change is consistent with TNFα (Fig. 6c–d). Thus, in mouse models and clinical specimens of tumors, TNFα increased in bevacizumab resistance tumors, and consistent with the number of M2b macrophage, as well as CCL1.

### Neutralizing TNFα strengthens the therapeutic effects of bevacizumab in vivo

**Fig. 7:**
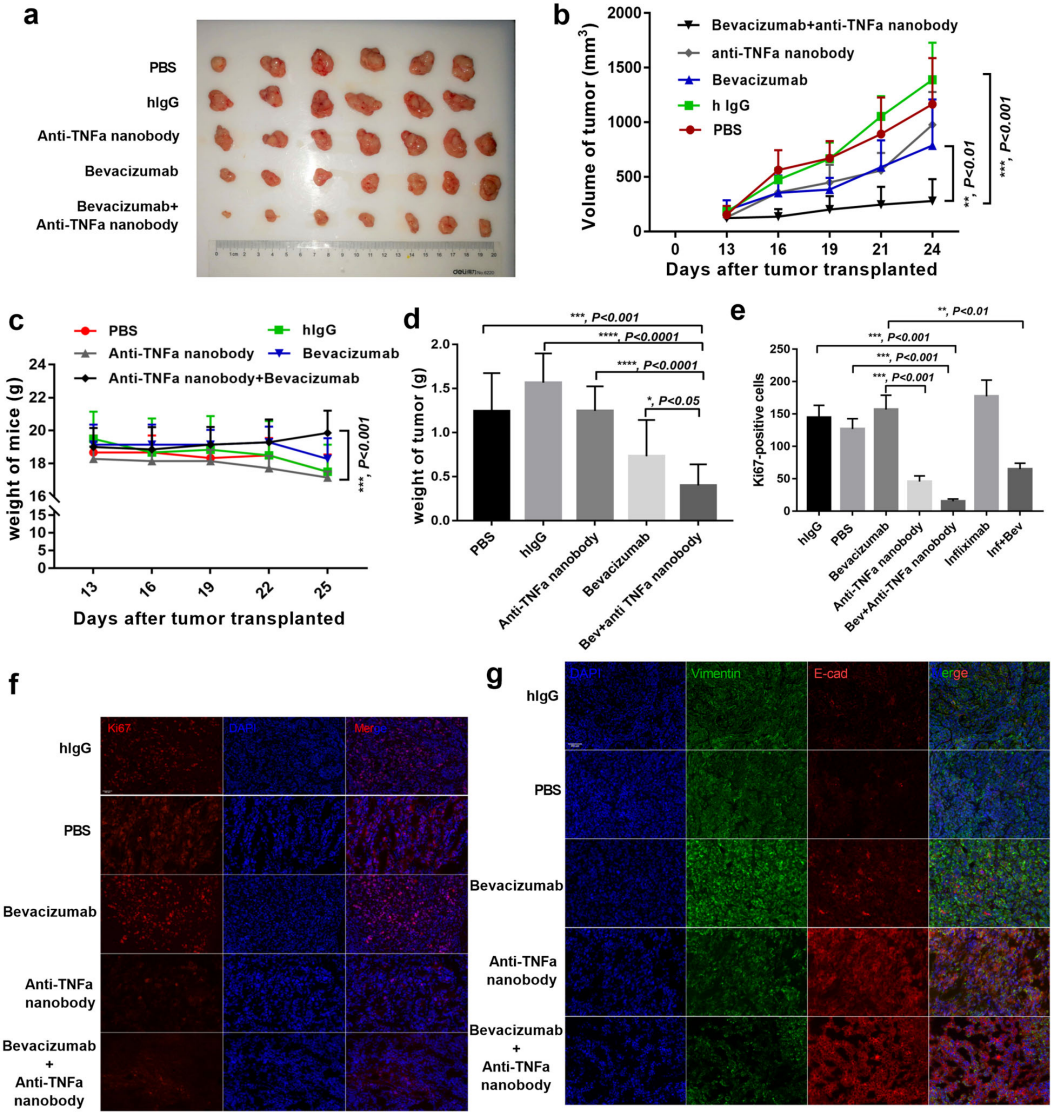
Neutralizing TNFα strengthens the therapeutic effects of bevacizumab in vivo. **a** Photos of MDA-MB-231-formed tumors under bevacizumab, anti-TNFα nanobody, alone or combination with both treatments. human IgG and PBS treated as control. **b** Tumor growth volume over time following subcutaneous transplant of tumor tissues into Balb/c nude mice. *n* = 7 for each condition. **P < 0.01; ***P < 0.001 by two-way ANOVA test. Error bars represent s.d. **c** Weight of mice over time following subcutaneous transplant of tumor tissues into Balb/c nude mice. **P < 0.01; ***P < 0.001 by two-way ANOVA test. Error bars represent s.d. **d** Tumor weight from humanely killed mice of each condition. *P < 0.05; ***P < 0.001; ****P < 0.0001 by *t* test. Error bars represent s.d. **e**–**f** Quantitative evaluation and representative images of double staining for Ki67 and nucleus. Scale bar, 200 μm. ***P < 0.001; ****P < 0.0001 by *t* test. Error bars represent s.d. **g** Representative images of tumor sections from mice treated with PBS, human IgG, bevacizumab, anti-TNFα nanobody, alone or combination with both treatments. Vimentin was co-stained with E-cadherin and nucleus. Scale bar, 200 μm.

Having confirmed the high concentration of TNFα in bevacizumab-resistant breast cancer and inhibition of TNFα suppressed tumor migration and IDO1 expression in vitro, we treated MDA-MB-231-bearing mice with bevacizumab, anti-TNFα nanobody, both, or PBS. We confirmed that anti-TNFα nanobody and infliximab could bind mouse TNFα (Supplementary Fig. 1c–d). Compared with those treated with bevacizumab alone, tumors treated with both bevacizumab and anti-TNFα nanobody exhibited significant tumor growth suppression (Fig. 7a–d). Ki67 expression in the tumors receiving bevacizumab combined with TNFα-neutralizing antibodies significantly decreased compared with levels in the bevacizumab group (Fig. 7e–f). We next investigated whether the combination of bevacizumab and anti-TNFα nanobody could inhibit the tumor epithelial–mesenchymal transition (EMT), which is an important indicator of tumor metastasis. Thus, we co-stained tumor samples for E-cadherin and vimentin, as the loss of the former by cancer cells is the main feature of the EMT. Vimentin was reduced dramatically in the combination treatment group (Fig. 7g). These data support the hypothesis that blocking the TNFα increase during bevacizumab administration enhances the therapeutic effects of anti-angiogenesis therapy in vivo.

## Discussion

Therapeutic mAbs have become important tools in contemporary cancer therapy and comprise a rapidly expanding class of drugs. However, mAb immunotherapy is also susceptible to both intrinsic and acquired resistance. Moreover, many mechanisms of resistance induced by Fc-dependent effector functions. FcγRs, especially CD16 and CD32a, mediate trastuzumab-dependent tumor phagocytosis by human macrophages, which converts macrophages into an immunosuppressive phenotype^13,37^. Although the ADCC and CDC mediated by antibodies play important roles in adaptive immunity, previous studies and our own research have shown the binding of therapeutic antibodies to macrophages in the tumor microenvironment though the Fc region to limit the efficacy of those antibodies. Both trastuzumab and rituximab, two IgG1 antibodies widely used in clinical cancer therapy, activate FcγRIII or FcγRII with their Fc domains and cause similar inhibitory effects^13,38^. Of the IgGs, IgG3 and IgG1 antibodies have the strongest affinity to FcγRs, so restricting the binding of antibodies to macrophages without affecting ADCC and CDC is an urgent problem to be solved.

It is now well established that tumor microenvironment contributes to drug resistance and therapy failure. Tumor-associated macrophages exhibit protumor functions involved in the suppression of immunity and re-establishment of homeostasis. Impeding macrophage M2b polarization by blockade of Notch1 signaling ameliorate murine lupus in the SLE murine^14^. Beyond all that, M2b macrophage has a number of immunosuppressive effects of in tumor progression. IgG4 redirects M2a macrophages to an M2b-like immunosuppressive phenotype^39^. Moreover, M2b secreted CCL1 are known to be Treg chemoattractant inducing suppression of host anti-tumor immunity^40^. As such, M2b macrophage are pervasively thought to have a protumor effect.

Considering originally potential mechanisms of TNFα induced tumor necrosis, we demonstrate that TNFα has protumor activity under bevacizumab administration in the tumor microenvironment. Tumor necrosis requires a high concentration of TNFα, which is often accompanied by high toxicity^41^. However, TNFα in the tumor microenvironment was on the level of ng^35^. TNFα is more susceptible to other excreted factors such as VEGF owing to the high complexity of the tumor microenvironment^36^. What’s more, diverse stages of tumor development lead to different effects of macrophage^18^, the major source of TNFα. Proinflammatory M1 macrophages gradually transform into protumor M2 macrophages during tumor development^42^, and TNFα concentration constantly evolves throughout the process.

Going forward, TLR4 was considered activation during bevacizumab administration and we proved it by treated RAW264.7 with LPS and bevacizumab in vitro. Although we found that HMGB1 was increased expressed in bevacizumab resistance tumor in vivo, a link between HMGB1 and TLR4 still unproved. Since apart from HMGB1, a multitude of endogenous ligands have been proposed to activate TLR4, any one or any combination of which could underlie the observed phenotypes in vivo. It will be important to clarify this issue.

In conclusion, we demonstrated the mechanism of bevacizumab resistance with a unique perspective, revealing the disadvantages of the inflammatory microenvironment created during tumor treatment. Our study offers a theoretical basis for the clinical treatment of cancer by inhibiting TNFα and provides a new strategy for overcoming clinical resistance to bevacizumab.

## Supplementary information

Supplementary Figure legends

Supplementary Figure 1

## References

[1] Macdonald D A JM (2016). Aflibercept exhibits VEGF binding stoichiometry distinct from bevacizumab and does not support formation of immune-like complexes. Angiogenesis.

[2] Harry, D. et al. Bevacizumab added to neoadjuvant chemotherapy for breast cancer. *N. Engl. J. Med.* 366 (2012). 10.1056/NEJMoa1111097 PMC3401076 22276821

[3] von Minckwitz G (2012). Neoadjuvant chemotherapy and bevacizumab for HER2-negative breast cancer. N. Engl. J. Med..

[4] Barton MK (2014). Bevacizumab in neoadjuvant chemotherapy increases the pathological complete response rate in patients with triple-negative breast cancer. CA Cancer J. Clin..

[5] Kong DH, Kim MR, Jang JH, Na HJ, Lee S (2017). A review of anti-angiogenic targets for monoclonal antibody cancer therapy. Int. J. Mol. Sci..

[6] Castro BA (2017). Macrophage migration inhibitory factor downregulation: a novel mechanism of resistance to anti-angiogenic therapy. Oncogene.

[7] Jahangiri A (2013). Gene expression profile identifies tyrosine kinase c-Met as a targetable mediator of antiangiogenic therapy resistance. Clin. Cancer Res..

[8] Mitsuhashi A (2015). Fibrocyte-like cells mediate acquired resistance to anti-angiogenic therapy with bevacizumab. Nat. Commun..

[9] Li B (2019). The kinetic changes of systemic inflammatory factors during bevacizumab treatment and its prognostic role in advanced non-small cell lung cancer patients. J. Cancer.

[10] Ramezani S, Vousooghi N, Joghataei MT, Chabok SY (2019). The role of kinase signaling in resistance to bevacizumab therapy for glioblastoma multiforme. Cancer Biother. Radiopharm..

[11] Horikawa N (2020). Anti-VEGF therapy resistance in ovarian cancer is caused by GM-CSF-induced myeloid-derived suppressor cell recruitment. Br. J. Cancer.

[12] Jetten N (2014). Anti-inflammatory M2, but not pro-inflammatory M1 macrophages promote angiogenesis in vivo. Angiogenesis.

[13] Su S (2018). Immune checkpoint inhibition overcomes ADCP-induced immunosuppression by macrophages. Cell.

[14] Zhang W, Xu W, Xiong S (2010). Blockade of Notch1 signaling alleviates murine lupus via blunting macrophage activation and M2b polarization. J. Immunol..

[15] Rhee I (2016). Diverse macrophages polarization in tumor microenvironment. Arch. Pharm. Res..

[16] Liu RX (2015). Chemokine (C-X-C motif) receptor 3-positive B cells link interleukin-17 inflammation to protumorigenic macrophage polarization in human hepatocellular carcinoma. Hepatology.

[17] Rőszer T (2015). Understanding the mysterious M2 macrophage through activation markers and effector mechanisms. Mediators Inflamm..

[18] Asai A (2017). Host antitumor resistance improved by the macrophage polarization in a chimera model of patients with HCC. Oncoimmunology.

[19] Yan J (2013). Inactivation of BAD by IKK inhibits TNFalpha-induced apoptosis independently of NF-kappaB activation. Cell.

[20] Grinberg-Bleyer Y (2017). NF-kappaB c-rel is crucial for the regulatory T cell immune checkpoint in cancer. Cell.

[21] Schioppa T (2011). B regulatory cells and the tumor-promoting actions of TNF-alpha during squamous carcinogenesis. Proc. Natl Acad. Sci. USA.

[22] Bigatto V (2015). TNF-alpha promotes invasive growth through the MET signaling pathway. Mol. Oncol..

[23] Johnston DA, Dong B, Hughes CC (2009). TNF induction of jagged-1 in endothelial cells is NFkappaB-dependent. Gene.

[24] Bowers E (2018). Granulocyte-derived TNFalpha promotes vascular and hematopoietic regeneration in the bone marrow. Nat. Med..

[25] Smith MP (2014). The immune microenvironment confers resistance to MAPK pathway inhibitors through macrophage-derived TNFalpha. Cancer Discov..

[26] Banzola I (2018). Expression of indoleamine 2,3-dioxygenase induced by IFN-gamma and TNF-alpha as potential biomarker of prostate cancer progression. Front. Immunol..

[27] Wardhani LO (2018). Expression of the IDO1/TDO2-AhR pathway in tumor cells or the tumor microenvironment is associated with MCPyV status and prognosis in Merkel cell carcinoma. Hum. Pathol..

[28] Wang H (2018). Functional role of kynurenine and aryl hydrocarbon receptor axis in chronic rhinosinusitis with nasal polyps. J. Allergy Clin. Immunol..

[29] Xue Y (2018). Indoleamine 2,3-dioxygenase expression regulates the survival and proliferation of Fusobacterium nucleatum in THP-1-derived macrophages. Cell Death Dis..

[30] Muller AJ, DuHadaway JB, Donover PS, Sutanto-Ward E, Prendergast GC (2005). Inhibition of indoleamine 2,3-dioxygenase, an immunoregulatory target of the cancer suppression gene Bin1, potentiates cancer chemotherapy. Nat. Med..

[31] Zhong WQ (2015). M2-polarized macrophages in keratocystic odontogenic tumor: relation to tumor angiogenesis. Sci. Rep..

[32] Biswas SK, Mantovani A (2010). Macrophage plasticity and interaction with lymphocyte subsets: cancer as a paradigm. Nat. Immunol..

[33] Kaneda MM (2016). PI3Kgamma is a molecular switch that controls immune suppression. Nature.

[34] Covell DG (1986). Pharmacokinetics of monoclonal immunoglobulin d, F(ab′)2, and Fab′ in mice. Cancer Res..

[35] Ji X (2017). Neutralization of TNFalpha in tumor with a novel nanobody potentiates paclitaxel-therapy and inhibits metastasis in breast cancer. Cancer Lett..

[36] Guadagni F (2007). Review. TNF/VEGF cross-talk in chronic inflammation-related cancer initiation and progression: an early target in anticancer therapeutic strategy. Vivo.

[37] Rittirsch D (2009). Cross-talk between TLR4 and FcgammaReceptorIII (CD16) pathways. PLoS Pathog..

[38] Roghanian A (2015). Antagonistic human FcgammaRIIB (CD32B) antibodies have anti-tumor activity and overcome resistance to antibody therapy in vivo. Cancer Cell.

[39] Bianchini R (2018). IgG4 drives M2a macrophages to a regulatory M2b-like phenotype: potential implication in immune tolerance. Allergy.

[40] Brambilla P (2014). Increased M1/decreased M2 signature and signs of Th1/Th2 shift in chronic patients with bipolar disorder, but not in those with schizophrenia. Transl. Psychiatry.

[41] Wu Y, Zhou BP (2010). TNF-alpha/NF-kappaB/Snail pathway in cancer cell migration and invasion. Br. J. Cancer.

[42] Chanmee T, Ontong P, Konno K, Itano N (2014). Tumor-associated macrophages as major players in the tumor microenvironment. Cancers.

