# Supplementary material for: Tumor necrosis factor α inhibition overcomes immunosuppressive M2b macrophage-induced bevacizumab resistance in triple-negative breast cancer
Source: Cell Death Dis. 2020 Nov 19;11(11):993. doi: 10.1038/s41419-020-03161-x (PMC7678839; doi:10.1038/s41419-020-03161-x)
Supplement: Supplementary file 1 — Supplementary Figure legends [file 41419_2020_3161_MOESM1_ESM.docx]

**Supplementary Figure. 1**

**a**, Binding analysis of bevacizumab, F(ab')2 of bevacizumab, human IgG and PBS with mouse VEGF. The concentration of pre-coated mouse VEGF was 1 μg/ml. ***, P<0.01, ****, P<0.0001* by t-test. Error bars represent s.d. **b**, Representative tube formation image of HUVEC cells treated with PBS, human IgG, bevacizumab and F(ab’)2 of bevacizumab in the with or without of mouse VEGF. Scale bar, 200μm. **c**, Binding analysis of anti-TNFα nanobody, infliximab, human IgG and PBS with mouse TNFα. The concentration of pre-coated mouse TNFα was 1 μg/ml. *****, P<0.0001* by t-test. Error bars represent s.d. **d**, Different concentration of Infliximab, anti-TNFα nanobody or human IgG (h IgG) were mixed with 10ng/ml TNFα and then added to L929 cells. Finally, cell survival was measured by MTT and PBS treated cells were control. ***, P<0.01, ***, P<0.001* by t-test. Error bars represents.d.
