# Supplementary figures and images for: Tumor necrosis factor α inhibition overcomes immunosuppressive M2b macrophage-induced bevacizumab resistance in triple-negative breast cancer
Source: Cell Death Dis. 2020 Nov 19;11(11):993. doi: 10.1038/s41419-020-03161-x (PMC7678839; doi:10.1038/s41419-020-03161-x)

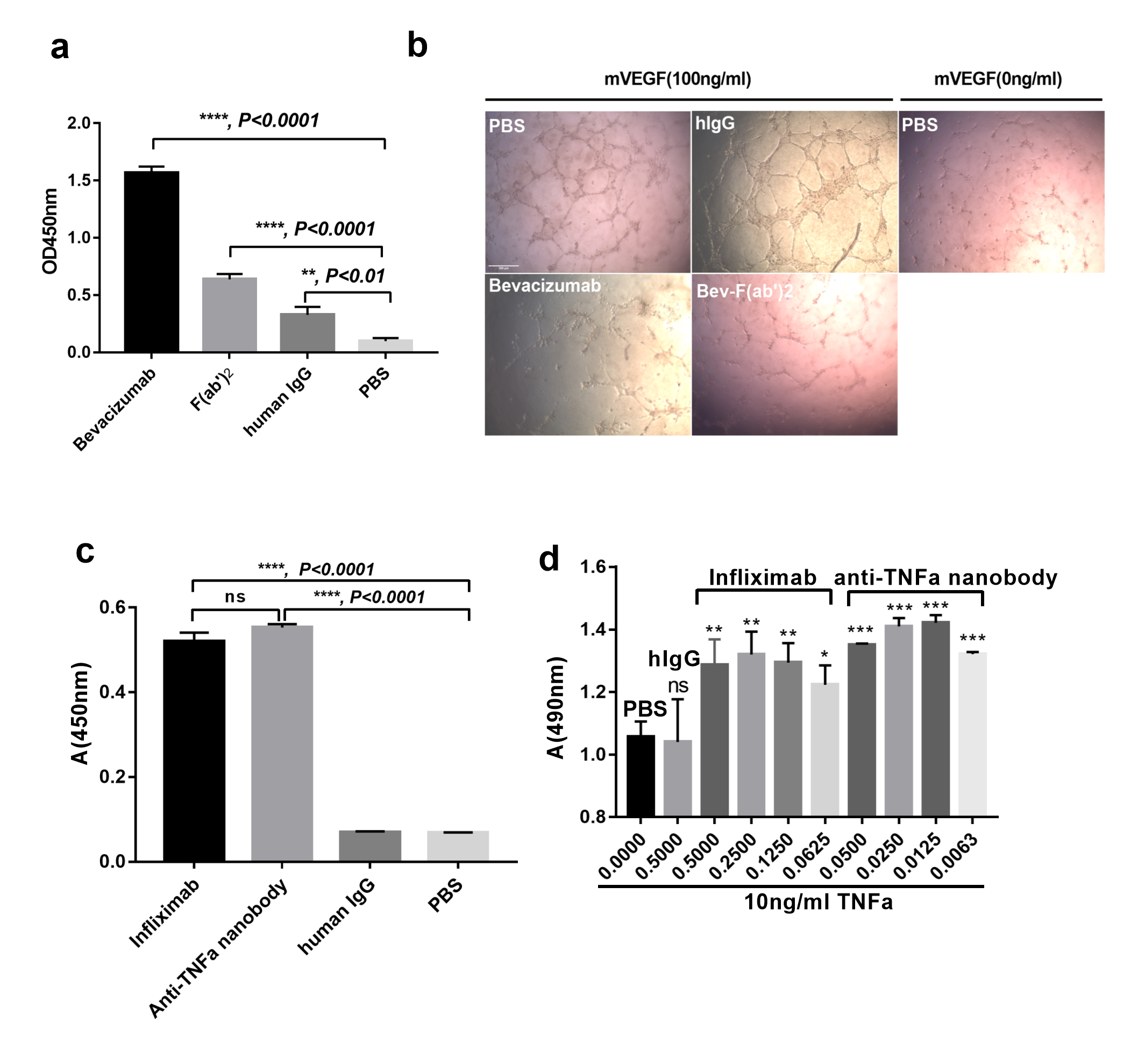

Supplement: Supplementary file 2 — Supplementary Figure 1 [file 41419_2020_3161_MOESM2_ESM.tif]
